# NCS 613, a Potent PDE4 Inhibitor, Displays Anti-Inflammatory and Anti-Proliferative Properties on A549 Lung Epithelial Cells and Human Lung Adenocarcinoma Explants

**DOI:** 10.3389/fphar.2020.01266

**Published:** 2020-08-18

**Authors:** Issaka Yougbare, Lazare Belemnaba, Caroline Morin, Abdurazzag Abusnina, Yannick F. Senouvo, Thérèse Keravis, Claire Lugnier, Eric Rousseau

**Affiliations:** ^1^Le Bilarium, Department of Physiology and Biophysics, Faculty of Medicine and Health Sciences, Université de Sherbrooke, Sherbrooke, QC, Canada; ^2^UMR CNRS 7213, Biophotonics and Pharmacology Laboratory, Faculty of Pharmacy, University of Strasbourg, Illkirch, France; ^3^Institute of Physiology, FMTS-EA 3072, Faculty of Medicine, University of Strasbourg, Strasbourg, France; ^4^Department of Obstetrics and Gynecology, Faculty of Medicine and Health Sciences, Université de Sherbrooke, Sherbrooke, QC, Canada

**Keywords:** PDE4 inhibitor, cAMP signaling, inflammation, proliferation, human lung adenocarcinoma

## Abstract

Chronic inflammation is a deleterious process occurring in several pulmonary diseases; it is a driving force promoting tumorigenesis. By regulating local cyclic nucleotide concentration, cyclic nucleotide phosphodiesterases (PDE) govern important biological processes, including inflammation and proliferation. The aim of this study was to investigate the anti-inflammatory and anti-proliferative effects of NCS 613, a specific PDE4 inhibitor, on TNFα-treated human lung adenocarcinoma cell line (A549) and on human lung adenocarcinoma explants. PDE4 isoforms and inflammatory pathways mediated by p38 MAPK, ERK1/2, and IκBα were analyzed by Western blot and immunostainings. Proliferation were performed using [^3^H]-thymidine incorporation under different experimental conditions. TNFα-stimulation increased p38 MAPK phosphorylation and NF-κB translocation into the nucleus, which was abolished by NCS 613 treatment. Concomitantly, NCS 613 restores IκBα detection level in human adenocarcinoma. An IC_50_ value of 8.5 μM was determined for NCS 613 on anti-proliferative properties while ERK1/2 signaling was down-regulated in A549 cells and lung adenocarcinoma explants. These findings shed light on PDE4 signaling as a key regulator of chronic inflammation and cancer epithelial cell proliferation. It suggests that PDE4 inhibition by NCS 613 represent potential and interesting strategy for therapeutic intervention in tackling chronic inflammation and cell proliferation.

## Introduction

**Figure 1:**
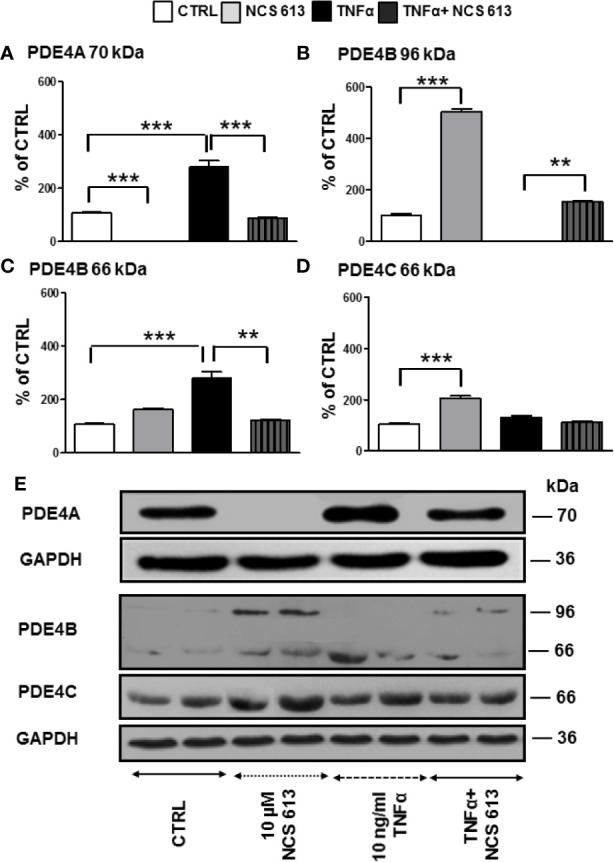
Detection of PDE4A, PDE4B, and PDE4C expressions in control and TNFα-stimulated A549 cells following NCS 613 treatment. A549 cells were stimulated with TNFα to induce inflammation and treated with NCS 613 for 48 h under the following experimental conditions: control, +10 μM NCS 613, + 10 ng/ml TNFα, and +TNFα + NCS 613. Relative protein expression levels were calculated following digitalization and image analysis from 3 independent experiments. **(A)** NCS 613 prevents and TNFα increases PDE4A 70 kDa overexpression; **(B)** NCS 613 induces PDE4B 96 kDa expression in A549 cell line; **(C)** TNFα stimulation induces PDE4B 66 kDa upregulation which is decreased with NCS 613 treatment; **(D)** PDE4C 66 kDa increases upon NCS 613 treatment; **(E)** representative immuno-reactive bands revealed by Western blot analysis (n=3). Unpaired Student’s *t*-test. Mean ± SEM. **p < 0.01 and ***p < 0.001.

Chronic inflammation, which is a deleterious process occurring in inflammatory respiratory diseases, is believed to be a tumor promoter in cancer induction (Lee et al., 2009; Balkwill and Mantovani, 2010; Phillips, 2020). Evidence suggests that recurrent injury and inflammation result in genetic alterations that predispose to lung cancer (Pikarsky and Ben-Neriah, 2006). NF-кB and p38 MAPK pathways play important role in human inflammatory diseases (COPD, rheumatoid arthritis…) and cancer (Koutras et al., 2009; Wong, 2009; Luangdilok et al., 2011). NF-кB dimers binding to target promoters lead to pro-inflammatory genes transcriptions and regulation of apoptosis and proliferation (Jeon et al., 2010). NF-κB activation is prevented by IκBα, an inhibitory protein which maintains NF-кB in an inactive state in the cytoplasm (Kamata et al., 2010). Downstream of surface membrane receptor activation, cyclic nucleotide phosphodiesterases (PDE) which encompass 11 families play a pivotal role in cAMP and cGMP signaling (Lugnier, 2006; Conti and Beavo, 2007). These enzymes hydrolyze cyclic nucleotides as a feedback mechanism for rapidly returning local nucleotide concentration to basal levels (Keravis et al., 2012; Maurice et al., 2014; Baillie et al., 2019; Lugnier et al., 2020). By regulating local cyclic nucleotides levels, PDEs contribute to their compartmentalization and govern number of biological processes, including inflammation and proliferation (Growcott et al., 2006; Sosroseno and Sugiatno, 2008, Abusnina et al., 2011a; Abusnina et al., 2011b; Alhosin, et al., 2011; Keravis, et al., 2011, Yougbare et al., 2013; Li et al., 2018; Baillie et al., 2019). Specific cAMP hydrolyzing phosphodiesterases (PDE4) have been shown to be involved in the control of inflammatory responses (Houslay, 2010). Inflammation is strongly correlated to lower intracellular cAMP levels. PDE4 implication has been reported in lung diseases related to chronic inflammation such as smoking-induced lung injury, fibrosis, asthma and COPD (Dunkern et al., 2007; Udalov et al., 2010; Diamant and Spina, 2011; Yougbare et al., 2014; Phillips, 2020). Inhibition of PDE4 activity causes elevation of intracellular cAMP levels and subsequent downregulation of a variety of inflammatory cell signaling (Yougbare et al., 2011). NCS 613 is a potent PDE4 inhibitor, which is more selective for the PDE4C subtype (IC_50_ = 1.4 nM) (**Figure 1E**). Compared other PDE4 inhibitors such as pentoxifylline and denbufylline, NCS 613 was the most potent and effective molecule in inhibiting both basal and LPS-induced TNFα secretion from leucocytes of lupus patients (Keravis et al., 2012). We previously showed that *in vivo* administration of NCS 613, a new and potent PDE4 inhibitor, reduced neutrophil recruitment in LPS-treated mouse bronchi and exhibits anti-inflammatory effects by decreasing TNFα secretion in guinea pig (Boichot et al., 2000). Interestingly, NCS 613 did not stimulate gastric acid secretion suggesting that this compound may produce gastrointestinal side effects (Boichot et al., 2000). Thus, PDE4 isozymes represent attractive targets for inflammation and cancer (Keravis et al., 2012; Li et al., 2018; Baillie et al., 2019; Hsien Lai et al., 2020). The above observations prompted us to investigate whether or not cellular signaling involved in inflammation and proliferation processes may be prevented by PDE4 inhibition and local increase in cAMP concentration. This study aimed to investigate the anti-inflammatory and antiproliferative effects of NCS 613 on human lung adenocarcinoma cell line (A549) and cultured human lung adenocarcinoma explants.

## Material and Methods

### Materials

The study was approved by our institutional Ethics Committee (Protocol number CRC 05-088 S1R2). Human lung explants were obtained from 4 patients aged between 50-60 years undergoing surgery for lung carcinoma resection. Tissues samples were collected from fresh lobectomy and transported to the laboratory in physiological Krebs’ solution (Sosroseno and Sugiatno, 2008). Human lung adenocarcinoma cell line (A549 cells) was from Abcam (# ab7910). NCS 613 (patent FR0601958) was given by J.J. Bourguignon and C. Lugnier (Faculty of pharmacy, Strasbourg). PDE antibodies used for Western blot analysis and immuno-staining were from FabGennix Inc. Anti-Phospho and total ERK1/2, anti-IκBα, anti-p65-NF-κB, Anti-phospho, and total p38-MAPK were from Cell Signaling Technology. [^3^H]-thymidine (250 μCi) was obtained from New England Nuclear.

### A549 Cells and Human Lung Adenocarcinoma Explants Culture

#### TNFα Induced Cell Responses

The adenocarcinoma human alveolar basal epithelial cells, A549 cells (1x 10^5^), were allowed growth for 24 h in T-75 culture flask, as previously described (Giard et al., 1973), in RPMI culture medium supplemented with 0.3% penicillin (100 IU/ml) and streptomycin (0.1 mg/ml) in presence of 1% fetal bovine serum at 37°C under 5% CO_2_. After 6 h starving period (without fetal bovine serum), cells were stimulated with TNFα to induce inflammation and treated with NCS 613 for 48 h under the following experimental conditions: control, +10 μM NCS 613, + 10 ng/ml TNFα, and +TNFα + NCS 613.

#### Cell Proliferation Assay

A549 cells were also cultured in 24-well plate in the presence of increasing NCS 613 concentrations of 0, 1, 2.5, 5, 7.5, 10, and 30 μM for 48 h. [^3^H]-thymidine incorporation was performed in triplicate for a 24 h period with 1 μCi tritiated thymidine. Harvested cells were lysed with cold 20% Trichloroacetic Acid (TCA) followed by DNA precipitation with 96% ethanol on Whatman paper. Radioactivity was quantified with β-counter using 5 ml scintillation liquid. IC_50_ value for NCS 613 was determined on cell proliferation as assessed by [^3^H]-thymidine incorporation (Denton, 1998).

#### Explant Proliferation Assay

After removal of connective tissues, lung adenocarcinoma explants were cultured in 6-wells culture plates containing RPMI as previously described for pulmonary tissue culture (Guibert et al., 2005). Explants were cultured in either control condition or treated with 1, 3, or 10 μM NCS 613. Tissues and harvested cells were homogenized with a Polytron in Radio-Immuno-Precipitation Assay buffer (RIPA) and supernatant aliquots were stored at -80°C until use.

### Western Blot Analysis

Proteins (25 μg) from lung adenocarcinoma and A549 cells lysates were subjected to Western blot analysis. Briefly, protein samples were electrophoresed on 10% SDS polyacrylamide gels and electro-transferred onto Polyvinylidene Difluoride (PVDF) membranes. Immunodetection was carried out with the following antibodies: anti-PDE4A, anti-PDE4B, anti-PDE4C, anti-PDE4D, anti-IκBα, anti-p38 MAPK, anti-ERK1/2 and anti-GAPDH. Immobilized antigens were detected by chemiluminescence using horseradish peroxidase-conjugated secondary antibodies, an ECL kit and autoradiography films.

### Immuno-Cytochemistry

Smears were made from harvested A549 cells, delineated with Dako Pen and fixed in 4%.

Paraformaldehyde (PFA) overnight before immuno-staining. After rinsing in TBS buffered (50 mM Tris, 150 mM NaCl, pH 7.6) and permeabilization with 0.1% Triton in PBS, smears were saturated with PBS + 4% BSA for 1 h at room temperature. Immuno-stainings were performed using specific antibodies directed against PDE4B, PDE4C, and NF-κB, and subsequently revealed by secondary antibodies coupled to either Alexa-488 or Alexa-654 fluorescent probe. Cell nuclei were labeled with DAPI (1 μM). Slides were mounted using Vectashield mounting medium and observed with a Leica microscope at 40X and 60X magnifications. Images were captured at the same magnification and exposure time allowing fluorescence comparison between the various experimental conditions.

### Data Analysis and Statistics

Results are expressed as means ± S.E.M. with n indicating the number of experiments.

Statistical analyses were performed using a one-way ANOVA followed by a Bonferroni post-test. Differences were considered statistically significant when p<0.05.

## Results

### Effects of NCS 613 on PDE4B and PDE4C Expressions in TNFα-Treated A549 Cells

Quantitative analysis of Western blot results showed that NCS 613 prevents PDE4A 70 kDa expression in A549 cells while TNFα significantly increases it. In these cells, the specific PDE4 inhibitor overcomes the overexpression of PDE4A (**Figure 1A**). Furthermore, long term inhibition of PDE4 by NCS 613 leads to induction of PDE4B 96 kDa in adenocarcinoma A549 cell line (**Figure 1B**). PDE4B 96 kDa expression was undetectable following TNFα stimulation and its expression increased upon NCS 613 treatment (**Figure 1B**). Conversely, the PDE4B 66 kDa isoform was increased upon TNFα treatment (**Figure 1C**). The PDE4C 66 kDa isoform on the other hand was increased in presence of 10 μM NCS 613 (**Figures 1D, E**). However, we did not detect PDE4D isoforms by Western blot in A549 cell line. These results suggest that cAMP accumulation subsequent to PDE4 inhibition induces a shift in isoform transcription and biosynthesis. The data also revealed that TNFα stimulation significantly abolished PDE4B 96 kDa while it increased PDE4A 70 kDa and PDE4B 66 kDa detection in A549 cells, suggesting that these later isoforms could be involved in TNFα signaling (**Figures 1A, C**). As expected, NCS 613 treatment overcame the TNFα-induced PDE4A 70 kDa and PDE4B 66 kDa increases which return to control level, hence indicating that this compound can interfere with inflammation signaling pathways.

### Effects of NCS 613 on PDE4B and PDE4C Locations in A549 Cells

**Figure 2:**
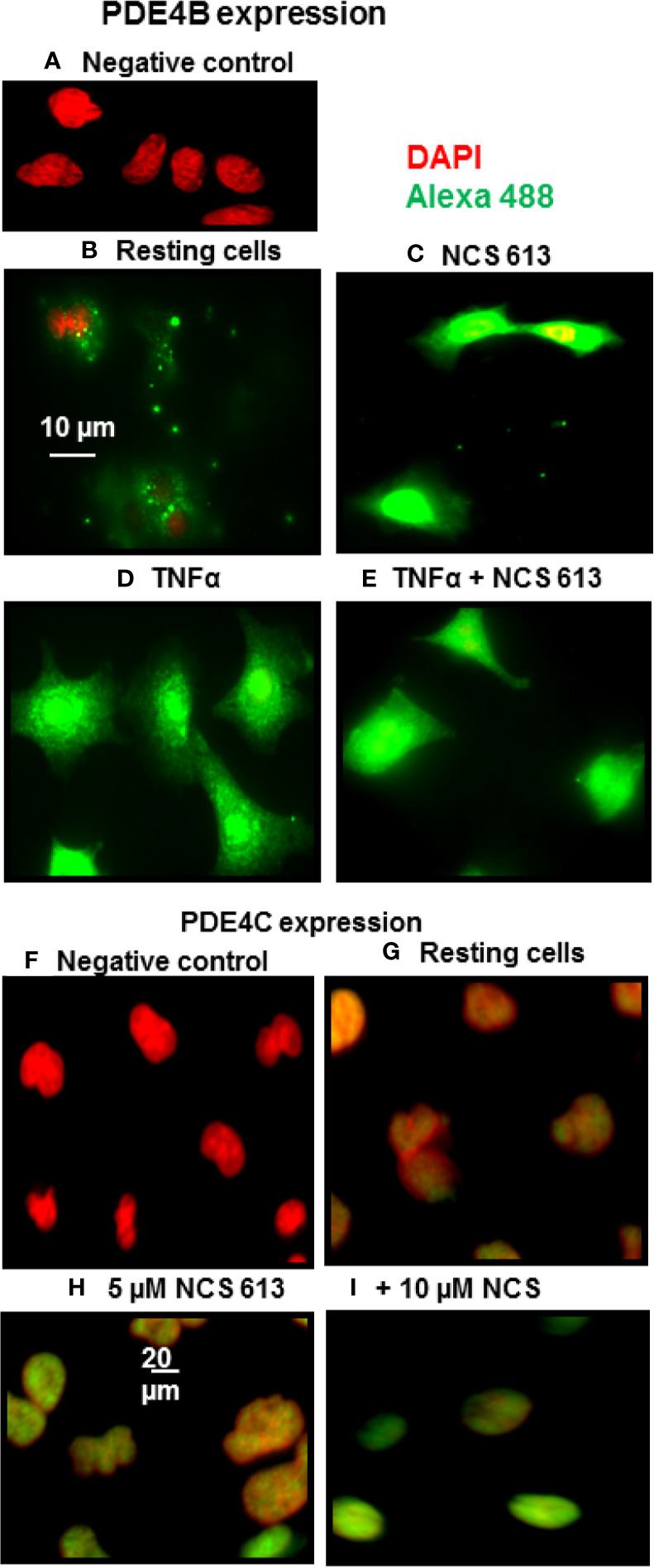
PDE4B and PDE4C immuno-cyto-staining. **(A–E)** Detection of PDE4B isoforms in A549 cells under control and TNFα treated conditions, in the absence or presence of NCS 613. **(A)** Negative control showing anti-PDE4B specificity; **(B)** PDE4B basal level in A549 cells; **(C)** NCS 613 increases PDE4B expression**; (D)** TNFα stimulation greatly increases PDE4B expression which is reduce by NCS 613 **(E)**. **(F–I)** Detection of PDE4C in A549 cells. **(F)** Negative control and **(G)** Basal PDE4C location; **(H)** and **(I)** NCS 613 treatments increase nuclear PDE4C in A549 cells. Data are representative of 3 independent experiments. Shown are merged images [x60 **(A–E)** and x40 **(F–I)** magnification] of DAPI staining (red) and anti-rabbit-Alexa Fluor 488 (green) recognizing primary anti-PDE4B or anti-PDE4C antibody (green).

Immuno-cytofluorescent staining confirms that NCS 613 treatment increased basal nuclear PDE4B and PDE4C expression (**Figure 2**). PDE4B 96 kDa could correspond to the nuclear isoform since this protein was only upregulated with NCS 613 treatment (**Figure 2C**). Immunostaining consistently showed that TNFα stimulation significantly increased PDE4B expression in A549 cells (**Figure 2D**). PDE4B 96 kDa was not expressed in A549 TNFα-stimulated cells (see **Figure 1B**), the 66 kDa isoform could correspond to the up-regulated cytoplasmic isoform. Interestingly, 10 μM NCS 613 treatment decreased PDE4B protein expression in TNFα-stimulated cells (**Figure 2E**). Complementary experiments were performed to delineate cellular location of PDE4C. Concomitant PDE4C detection using a specific monoclonal antibody and DAPI counter-staining demonstrates the nuclear localization of this isoform (**Figures 2F, G**). Lower panels show that NCS 613 treatments (5 and 10 μM) increased the detection of PDE4C isoform in A549 cells (**Figures 2 G–I**). Together these results indicate that NCS 613 treatment modulates the compartmentalized expression of PDE4 isoforms.

### Effects of NCS 613 on p38 MAPK Phosphorylation and NF-κB Location

**Figure 3:**
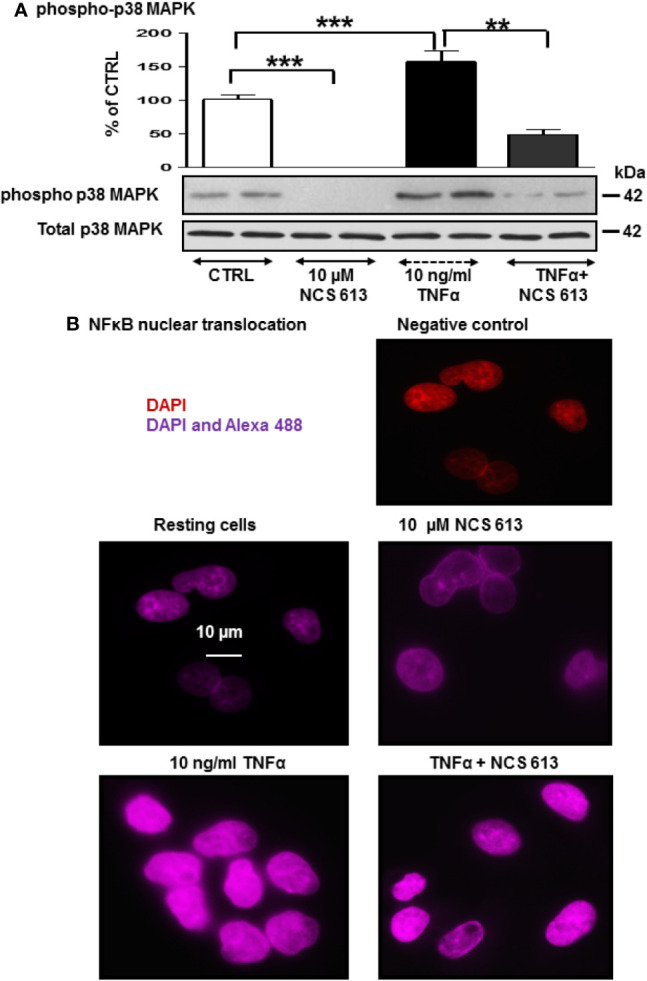
Effect of NCS 613 on p38 MAPK phosphorylation and NF-κB nuclear translocation upon TNFα stimulation. **(A)** TNFα increases and NCS 613 reduces p38 MAPK phosphorylation in A549 cells; **(B)** NCS 613 prevents NF-κB translocation into nucleus while TNFα stimulation greatly increases NF-κB p65-subunit translocation into nucleus. Shown are merged images (60x magnification) of DAPI staining and anti-mouse Alexa 654 recognizing primary p65-NF-κB antibody (n=3). Unpaired Student’s *t*-test. Mean ± SEM. **p < 0.01 and ***p < 0.001.

After 72 h culture, phospho-p38 MAPK was detected in both control and TNFα-treated A549 cells (**Figure 3A**), whereas NCS 613 treatment abolished p38 MAPK phosphorylation demonstrating a net effect of this compound. TNFα stimulation significantly increases p38 MAPK phosphorylation facilitating down-stream effectors activation. NCS 613 treatment abolishes the TNFα-induced p38 MAPK phosphorylation, while the total p38 MAPK expression level remains unchanged (**Figure 3A**). Since it has been reported that p38 MAPK phosphorylation induces NF-κB activation (Morin et al., 2008; Smith et al., 2010; Sigala et al., 2011), antibodies against the NF-κB p65-subunit were used to detect NF-κB translocation into the nucleus. NCS 613 treatment reduces NF-κB basal detection in these cells (**Figure 3B**). In contrast, TNFα stimulation enhances of NF-κB translocation into the nucleus, while NCS 613 treatment counteracted the effects of TNFα on p65-NF-κB translocation. Together, these results indicate that intracellular cAMP level, which is increased during NCS 613 treatment, modulates p38 MAPK phosphorylation and downstream nuclear translocation of p65-subunit of NF-κB.

### Anti-Inflammatory Effects of NCS 613 Treatment on IκBα Expression

**Figure 4:**
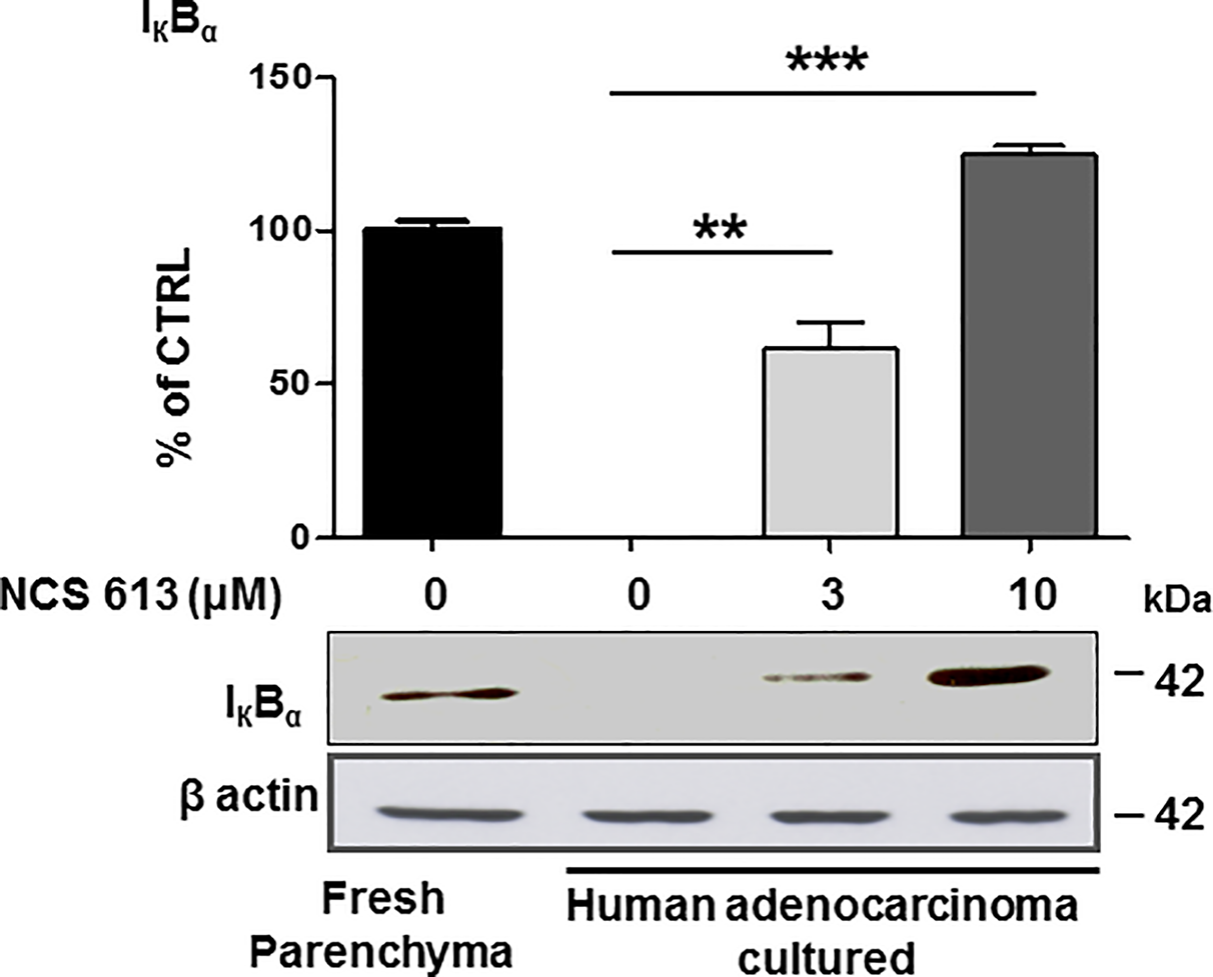
Concentration dependent effect of NCS 613 treatment on IκBα detection in adenocarcinoma explants. IκBα was detected in fresh human lung parenchyma. After a 72 h culture period, IκBα was undetectable in control adenocarcinoma explants suggesting a high inflammation level. Three and 10 µM NCS 613 treatment reestablished IκBα detection level (n= 4). Unpaired Student’s *t*-test. Mean ± SEM. **p < 0.01 and ***p < 0.001.

Increasing evidences have revealed that tissue inflammation is a recurrent component in cancer (Balkwill and Mantovani, 2010; Smith et al., 2010). To assess the putative anti-inflammatory effects of NCS 613, IκBα expression was used as a lung inflammation marker (lower detection indicates larger inflammation). IκBα was detected in fresh human lung parenchyma (positive control, **Figure 4**) whereas IκBα was undetectable in untreated (control) lung adenocarcinoma explants cultured for 72, which is correlated with an increased inflammation process. In contrast, NCS 613 (at 3 and 10 μM) rescued IκBα detection in a concentration-dependent manner, which could correlate with a lower inflammation status of the tissues.

### NCS 613 Displays Antiproliferative Effects Through ERK1/2 Inhibition

**Figure 5:**
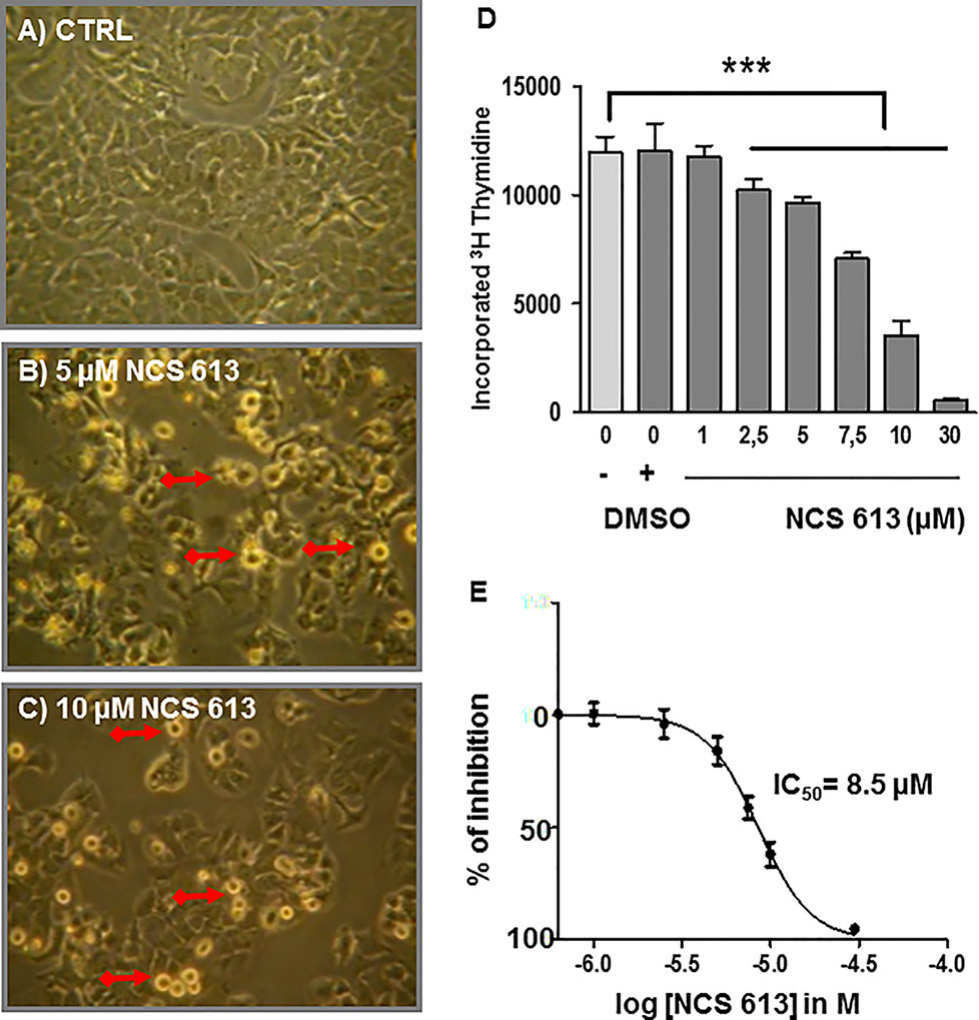
Anti-proliferative effect of NCS 613 on A549 cells. **(A)** Control: A549 cells display rapid proliferation rate toward confluency. **(B, C)** A549 cells treated with either 5 µM or 10 µM NCS 613 for 48 h results in lower density and higher apoptosis. Plates are representative of three independent experiments; **(D)** Bar histogram showing [^3^H]-thymidine incorporation into DNA. Gradual (2.5–30 µM) NCS 613 treatment significantly decreases A549 cells proliferation; **(E)** Concentration dependent inhibition curve displaying the antiproliferative effects of NCS 613 from which an IC_50_ value of 8.5 µM was calculated. Unpaired Student’s *t*-test. Mean ± SEM. ***p < 0.001.

**Figure 6:**
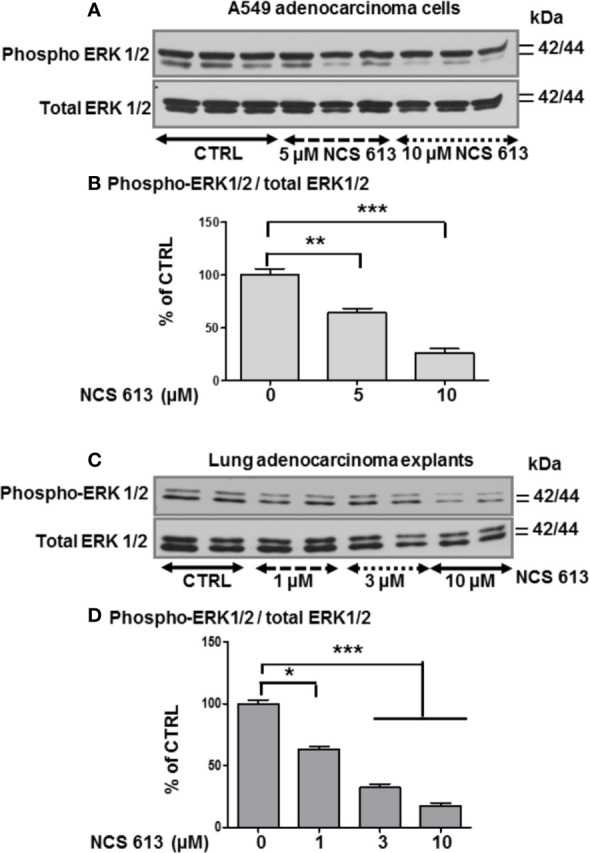
Effect of NCS 613 treatment on ERK1/2 phosphorylation. **(A, B)** Concentration dependent effect of NCS 613 on the phosphorylation level of ERK1/2 as a function of experimental conditions (n=3); **(C, D)** Concentration dependent effect of NCS 613 on phospho-ERK1/2 detection in cultured lung adenocarcinoma explants (n=4). Unpaired Student’s *t*-test. Mean ± SEM. *p < 0.05, **p < 0.01 and ***p < 0.001.

In the absence of treatment, A549 cells proliferate and become highly confluent (**Figure 5A**). Interestingly, NCS 613 treatment opposed this effect on cells proliferation and induced cells apoptosis (**Figures 5B, C**). DMSO up to 0.3 ‰, used as vehicle did not exhibit any toxicity toward A549 cells (**Figures 4D, E**). Of note, NCS 613 (2.5–30 μM) significantly decreased [^3^H]-thymidine incorporation, hence correlating with a lower cell proliferation rate. Moreover, inhibition curve displays an anti-proliferative effect of NCS 613 with an IC_50_ value of 8.5 μM. NCS 613 treatment reduces ERK 1/2 phosphorylation as well as total ERK 1/2 in A549 cells (**Figures 6A, B**) as well as in human lung adenocarcinoma explants (**Figures 6C, D**) attesting that this pathway is likely involved in the anti-proliferative effect of this compound. Several putative pathways involved in inflammatory responses and cell proliferation are likely modulated by NCS 613. As shown in the present results, TNFα induces PDE4B up-regulation in order to hydrolyze cAMP and activates p38 MAPK and NF-κB pathways, which in turn leads to IκBα degradation. NCS 613 on the other hand increases cAMP levels and thus prevents p38 MAPK phosphorylation. NCS 613 can also inhibit cell proliferation by a down-regulation of ERK1/2 signaling. The sustained inhibition of PDE4 by NCS 613 induces the synthesis of novel isoforms as a feedback mechanism to modulate cAMP levels.

## Discussion

Chronic inflammation is a hallmark of pulmonary diseases, which can lead to emphysema and airways hyperresponsiveness. In the present study, we were able to delineate the role of PDE4 in TNFα-induced inflammation in A549 cells in which PDE4A 70 kDa and PDE4B 66 kDa were significantly up-regulated but not PDE4C and PDE4D. This upregulation of PDE4 isoforms, downstream of TNFα-receptor activation, facilitates cAMP breakdown and activation of inflammatory effectors. Under these conditions, A549 cells consistently expressed higher cytoplasmic levels of PDE4B as revealed by immuno-staining. NCS 613 treatment, which inhibits PDE4 activity, restored intracellular cAMP levels, resulting in an opposite effect on inflammatory markers. In a previous report, LPS-induced TNFα secretion was shown to be significantly reduced in macrophages from bronchi-alveolar fluid of PDE4B-deficient mice (Jin et al., 2005). Comparing the effect rolipram, NCS 613 and RP 73401, or the cell permeable analogue N6 -2’-O-dibutyryladenosine 3’,5’-cyclic monophosphate (db- cAMP) on histamine release by mast cells from diabetic rats, we observed that these molecules led to a decrease of histamine secretion *in vitro* (Barreto et al., 2004). Importantly, the effectiveness of either NCS 613 or db cAMP in inhibiting antigen-induced degranulation is comparable in both normal and diabetic mast cells. Our current data suggest that the PDE4A 70 kDa and PDE4B 66 kDa isoforms are likely involved in TNFα signaling, which is consistent with the conclusion of a recent study showing that the induction of PDE4B is required for Toll-like receptor signaling in monocytes and macrophages (Jin et al., 2010).

Herein, the PDE4B 96 kDa increase in A549 cells by NCS 613 treatment, suggests that this isoform is likely involved in the regulation of cAMP metabolism. This is also supported by the upregulation of nuclear PDE4C in A549 cells. Moreover, other reports revealed that sustained increased in intracellular cAMP enhanced PDE4A/4B gene expressions in U937 cells as well as l PDE4B/4D in human myometrial cells (Dunkern et al., 2007). Interestingly, it was previously reported that PDE4B2 was upregulated by IL-1β at mRNA and protein levels from human myometrial cells through a prostaglandin E2- and cyclic adenosine 3’, 5’-monophosphate-dependent pathway (Oger et al., 2002). PDE4 activity and expression in cultured pulmonary microvascular endothelial cells from rat are activated by a PKA *in situ* phosphorylation in short-term and induced in long-term *via* increases of intracellular cAMP concentrations (Zhu et al., 2004). Our work showed the specificity and functions of PDE4B and PDE4C isoforms in which 96 kDa and 66 kDa proteins respectively regulate cAMP level in A549 cells. PDE4 compartmentalization in cells tightly regulates cAMP level underpinning a complex cAMP signaling network (Houslay, 2010; Yougbare et al., 2013). Two pathways involved in the typical inflammatory response were assessed in the present study. The stress kinase p38 MAPK is thought to regulate NF-κB translocation to nuclei (Moriyuki et al., 2009; Morin et al., 2010) and TNFα stimulation increases p38 MAPK phosphorylation. In addition increasing evidence suggested that PDE4 inhibitor are negative modulator of p38 phosphorylation (Kwak et al., 2005; Yougbare et al., 2011). NCS 613 totally abolishes p38 MAPK phosphorylation and reduces NF-κB translocation to the nucleus. Consequently, the use of NCS 613 opposes to p38 MAPK activation by stress stimuli (TNFα, LPS) and its phosphorylation leading to rapid activation of NF-κB (Morin et al., 2010). Consistent with these observations, NCS 613 treatment stabilizes IκBα binding to NF-κB and prevents nuclear translocation of the latter. Under pro-inflammatory conditions, IκBα is usually phosphorylated by IκK and then ubiquitinated, leading to its degradation into proteasomes, and lower cytoplasmic detection and NF-κB translocation to the nuclei (Houslay and Adams, 2010; Kamata et al., 2010). In cultured human lung adenocarcinoma explants IκBα was undetectable in control (untreated) conditions whereas NCS 613 was able to restore IκBα detection in a concentration-dependent manner. Thus, IκBα protection down regulates NF-κB activation which have important implications in lung cancer prevention and immunotherapy (Lin et al., 2010). Our current data suggest that NCS 613 may have potent anti-inflammatory properties in A549 cells and lung carcinoma explants.

Microscopic observation of A549 cells under specific PDE4 inhibitor treatment provides the first evidence that NCS 613 can also markedly affect cell proliferation. A549 cells death was observed with Trypan blue staining during cells count. Trypan blue, a vital stain used to selectively color dead cells in blue reveals A549 cells death mediated by NCS 613 treatment. Other compounds like CC-8075 and CC-8062 are PDE4 inhibitors that have been shown an anti-proliferative effect on various cell lines by increasing cAMP (Koutras et al., 2009; Mouratidis et al., 2009; Hsien Lai et al., 2020). The [^3^H]-thymidine incorporation proliferation assay confirms that NCS 613 decreases cell proliferation concomitantly with lower detection of inflammatory markers suggesting the contribution of the latter in cancer progression. Several studies have used mouse cancer models and have provided direct genetic evidence for the critical role of NF-κB in carcinogenesis (Pikarsky and Ben-Neriah, 2006). With an IC50 value of 8.5 μM on cell proliferation, NCS 613 reduces cell division and likely induces apoptosis in malignant cells, which is corroborated by the downregulation of phospho- ERK1/2 in A549 cells. This observation could be correlated to a recent finding suggesting a predominant role of PDE4B in controlling molecular pathophysiological processes involved in the proliferation of human lung fibroblasts (Selige et al., 2010). The mechanism by which cAMP induced apoptosis in A549 cells remains to be established.

## Conclusion

The present data highlight the role of PDE4B in controlling inflammatory responses and cell proliferation pathways. NCS 613 targets PDE4 and, indirectly, p38 MAPK as well as NF-κB and ERK1/2 signaling, leading to a down-regulation of inflammation in both the human A549 cell line and lung adenocarcinoma explants. NCS 613 thus represents a potent anti-inflammatory compound, which did not induce gastrointestinal side effects at 10 mg/kg i.v. (Boichot et al., 2000), which may be useful in the treatment of chronic inflammatory diseases and cancer prevention in pre-clinical studies. Further studies are needed to assess whether NCS 613 induces apoptosis in A549 and lung adenocarcinoma.

## Data Availability Statement

The raw data supporting the conclusions of this article will be made available by the authors, without undue reservation.

## Ethics Statement

The studies involving human participants were reviewed and approved by Protocol number CRC 05-088 S1R2. The patients/participants provided their written informed consent to participate in this study.

## Author Contributions

IY designed and performed all the experiments, analyzed and interpreted data, and prepared and revised the manuscript. LB, AA, and YS conducted some experiments and prepared the manuscript. CM and TK provided guidance for study design and data interpretation. CL contributed to study design and data interpretation, as well as manuscript preparation. As the principle investigator, ER supervised study design, data interpretation, and manuscript preparation.

## Funding

This work was supported by transition grants from the FMSS and CRCHUS. IY and CM were recipients of PhD-studentships from the European Doctorate College EDC (Strasbourg) and NSERC of Canada, respectively.

## Conflict of Interest

The authors declare that the research was conducted in the absence of any commercial or financial relationships that could be construed as a potential conflict of interest.
